# Diagnostic yield and adverse events of liver biopsy in intensive-care-unit patients: a multicenter retrospective observational cohort study

**DOI:** 10.1186/s13613-025-01533-0

**Published:** 2025-08-26

**Authors:** Mégane Charrier, Jean-Claude Lacherade, Lara Zafrani, Jérôme Hoff, Jean Reignier, Jean-Baptiste Lascarrou, Jean-François Mosnier, Emmanuel Canet

**Affiliations:** 1 Medical Intensive Care Unit, Nantes University Hospital, Nantes University, Nantes, France; 2 Medical Intensive Care Unit, District Hospital Center, La Roche-sur-Yon, France; 3 Medical Intensive Care Unit, AP-HP, Saint-Louis Hospital, University of Paris Cité, Paris, France; 4 Medical Intensive Care Unit, District Hospital Center, Saint-Nazaire, France; 5 Department of Pathology, Nantes University Hospital, Nantes University, Nantes, France; 6 ICU, Nantes University, Nantes University Hospital, Movement - Interactions– Performance Research Unit MIP, Nantes, 4334 UR France

**Keywords:** Liver biopsy, Intensive care unit, Cirrhosis, Hepatitis, Hemorrhage, Diagnostic yield

## Abstract

**Background:**

To investigate the adverse events, diagnostic yield, and therapeutic implications of liver biopsy in intensive-care-unit (ICU) patients.

**Methods:**

Retrospective observational multicenter cohort study. Consecutive adults admitted to any of the four participating ICUs in France between January 1, 2006 and March 1, 2023, and who had a liver biopsy during the ICU stay were included.

**Results:**

We included 139 patients (median age, 52 years; 69% male) biopsied via the transjugular (*n* = 97), percutaneous (*n* = 30), or laparoscopic (*n* = 1) route (missing data *n* = 11). The liver parenchyma was evaluable in 137/139 (99%) patients, who had 187 histological diagnoses in total. The pathological diagnoses matched the pre-biopsy diagnostic hypotheses in 83 (60%) patients. The most common were chronic or acute-on-chronic liver disease (*n* = 78, 56%), malignancy (*n* = 27, 19%), and infectious disease (*n* = 12, 9%). Among other diagnoses (*n* = 17, 12%), drug toxicity and biliary diseases predominated. The liver biopsy had therapeutic implications for 80 (58%) patients, among whom 66 (82%) received a new treatment, 7 (9%) were continued on empirically initiated treatment, and 7 (9%) were taken off the previous treatment. WHO grade 3–4 bleeding developed in 10 (7%) patients and was fatal in 2 patients. Higher severity scores, higher urea level, and absence of cirrhosis were associated with a greater risk of bleeding complications. Day-90 survival was not significantly different between the groups with vs. without therapeutic implications of the biopsy.

**Conclusions:**

In ICU patients, liver biopsy provides a wide range of diagnoses and guides treatment decisions. However, the risk of potentially fatal bleeding is a major concern. We identified risk factors for bleeding.

**Supplementary Information:**

The online version contains supplementary material available at 10.1186/s13613-025-01533-0.

## Background

Liver function test abnormalities are common in patients admitted to the intensive care unit (ICU) [1]. In a large prospective study, early liver dysfunction was independently associated with higher mortality [2]. The many factors that can cause liver function test abnormalities in critically ill patients have received limited research attention. Primary liver diseases such as cirrhosis, acute-on-chronic liver failure (ACLF), and viral hepatitis are common. Shock can induce ischemic hepatitis, with a massive increase in serum aminotransferase levels [3]. Other causes include sepsis [4], hematological and solid malignancies, macrophage activation syndrome [5], exposure to toxic substances [6], and auto-immune diseases [7]. Identifying the cause is important to guide treatment decisions.

Liver biopsy is a valuable diagnostic tool but can cause complications, notably bleeding. Blood biomarker assays, serological tests, and imaging studies are non-invasive methods that can obviate the need for liver biopsy [8]. However, their diagnostic performance has not been validated specifically in ICU patients and their results are not always conclusive, leaving liver biopsy as the reference diagnostic tool [9, 10, 11]. Whether the hemostasis impairments often seen in critically ill patients increase the risk of bleeding during liver biopsies has not been investigated [12].

The primary objective of this multicenter retrospective observational cohort study was to assess the diagnoses established by liver biopsy in ICU patients. The secondary objectives were to evaluate the safety and therapeutic consequences of liver biopsy.

## Methods

The study was approved by the ethics committee of the French Intensive Care Society (CE SRLF 23–085) on November 24, 2023. In accordance with French law on retrospective studies of anonymized healthcare data, informed consent was not required. This report complies with STROBE guidelines [13] (e-Table 1).

### Study design and population

Four ICUs (Nantes University Hospital, Saint-Louis University Hospital, La Roche-sur-Yon Hospital, and Saint-Nazaire Hospital) in France participated in the study. We searched the databases of the four ICUs to identify adults (age > 18 years) who were admitted between 1 January, 2006, and 1 March, 2023. We then selected those patients who had a liver biopsy by looking for the International Classification of Diseases (ICD-10) code Z139 (liver biopsy) and the codes for liver biopsy in the French classification of healthcare interventions (HLHJ006, HLHB001, HLHH002, HLHH005, HLHH006, HLHH007, HLHJ003, and HLHJ005). Exclusion criteria were liver abscess puncture or drainage, pancreatic necrosis, collection in the gallbladder or any other abdominal site, and unavailability of the liver-biopsy pathology report. The principal investigator (MC) reviewed each medical file to check that all inclusion criteria and none of the exclusion criteria were met. For patients who had more than one ICU stay during the study period, only the first admission was considered. All aspects of liver biopsy procedure (indication, route, management of hemostasis impairment and monitoring) were at the discretion of the physician in charge.

### Data collection and outcomes

For each patient, the data reported in the tables were extracted from the ICU records and entered by the principal investigator (MC) into a standardized electronic case-report form. Comorbidities were recorded using the Charlson Comorbidity Index [14] and critical-illness severity using the Simplified Acute Physiology Score version II (SAPS II) [15] and Sequential Organ Failure Assessment (SOFA) score [16]. In patients with a chronic liver disease, the Child-Pugh score [17], Model for End-stage Liver Disease (MELD) score [18, 19], and Chronic Liver Failure Consortium Organ Failure score (CLIF-C OF)

 [20] were determined and the ACLF grade [21] was collected. The Maddrey score was computed in patients with a clinical suspicion of severe acute alcoholic hepatitis [22].

The severity of bleeding complications was classified according to the Modified WHO Bleeding Scale [23] (e-Appendix 2). Transfusions of blood products during the 48 h following the liver biopsy were collected. The potential link between bleeding complications and the liver biopsy was evaluated based on the medical notes, identification of the liver as the source of bleeding by imaging, computed tomography or ultrasound findings, endoscopic procedures, and absence of other sources of bleeding.

The pathological reports were reviewed to collect the prevalence of evaluable samples, defined as containing liver parenchyma; the final pathological diagnoses; and treatment decisions (initiation, continuation or discontinuation of a treatment) based on the liver biopsy results.

Day-90 vital status was recorded for all patients.

### Statistical analysis

Descriptive statistics were computed for the baseline features in the overall population. Qualitative data were described as number (percentage) and compared using the chi-square test or, if necessary, Fisher’s exact test. Quantitative data were described as mean ± SD if normally distributed and as median [interquartile range] otherwise; comparisons were with Student’s *t* test and the Mann-Whitney test, respectively. Missing data were counted and reported in tables.

The statistical analyses were done using the R statistics program, version 3.5.0 (R Foundation for Statistical Computing, Vienna, Austria; www.R-project.org/). *P* values lower than 0.05 were taken to indicate significant differences.

### Results

### Study population

**Fig. 1:**
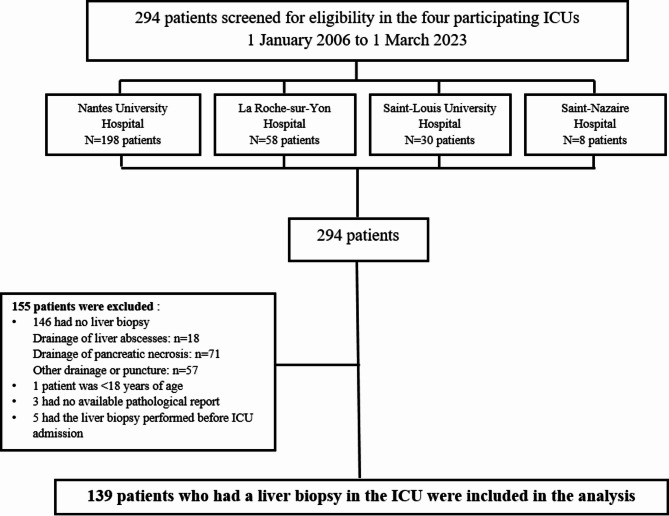
Flowchart of the study. ICU: intensive care unit

Figure 1 is the flow chart. We included 139 patients who had a liver biopsy in the participating ICUs. Table 1 reports their main characteristics. Males in their fifties predominated and excessive alcohol consumption was common. Known cirrhosis was present in 29 (21%) patients and cirrhosis was suspected at ICU admission in an additional 55 (40%) patients based on physical findings and laboratory tests. Among the 84 patients with suspected or confirmed cirrhosis, 76 (91%) had a Child score C10-C15; the median MELD and CLIF-C-OF scores were 26 [23–29] and 11 [10–13], respectively; among the 84 (60%) patients with ACLF, 19 (23%) had grade 2 and 22 (26%) had grade 3 (missing data *n* = 23). In the 77 (55%) patients with suspected severe acute alcoholic hepatitis, the median Maddrey score was 65 [45–74]. Immunodeficiency was present in 44 (32%) patients and was usually related to malignancy. Many patients required invasive mechanical ventilation and vasopressors, whereas renal replacement therapy was less often used.

**Table 1:** Characteristics of the 139 patients who had a liver biopsy in the intensive care unit

Patient characteristics	*N* (%) or Median [IQR]	*n* missing data
Age, years	52 [44–59]	0
Males	96 (69)	0
Body mass index (kg/m²)	25 [22–28]	26 (19)
Current smoker	61 (44)	31 (22)
Excessive alcohol consumption	81 (58)	26 (19)
Charlson Comorbidity Index	4 [3–6]	0
Immunodeficiency	44 (32)	1 (1)
Hematological malignancy	19 (14))	
Solid cancer	16 (12)	
Other^*a*^	9 (6)	
Known history of cirrhosis	29 (21)	
**ICU admission from the ED**	78 (56)	
**Reason for ICU admission**		0
Altered consciousness	24 (17)	
Gastrointestinal bleeding	26 (19)	
Respiratory failure	17 (12)	
Sepsis	35 (25)	
Acute kidney injury	4 (3)	
Multiple organ failure	18 (13)	
Other^*b*^	15 (11)	
**Severity scores at ICU admission**		
SOFA score	8 [5–11]	9 (7)
SAPS II	50 [34–60]	5 (4)
**Liver tests on the day of biopsy**		
Total bilirubin, µmol/L	159 [74–258]	21 (15)
AST, IU/L	138 [86–287]	20 (14)
ALT, IU/L	62 [37–159]	20 (14)
Alkaline phosphatase, IU/L	156 [103–281]	24 (17)
GGT, IU/L	204 [109–420]	25 (18)
**Organ support during the ICU stay**		
Invasive mechanical ventilation	92 (66)	0
Vasopressors	79 (57)	6 (4)
Renal replacement therapy	40 (29)	0
Extracorporeal membrane oxygenation	2 (1)	0

AST: aspartate aminotransferase; ALT: alanine aminotransferase; ED: emergency department (including pre‑hospital emergency medical service); GGT: gamma-glutamyl transferase; ICU: intensive care unit; SAPS II: Simplified Acute Physiology Score version II; SOFA: Sequential Organ Failure Assessment

^a^AIDS, corticosteroid therapy, immunosuppressive treatments

^b^Hematological disease (*n* = 6), liver disease (*n* = 4), cardiac arrest (*n* = 3) endocrinological disease (*n* = 1), hypovolemic shock (*n* = 1)

### Liver biopsies

The reasons for liver biopsy were suspected severe acute alcoholic hepatitis (*n* = 77, 55%), investigation of a focal liver lesion (*n* = 16, 12%), clinical suspicion of hematological malignancy and/or macrophage activation syndrome (*n* = 14, 10%), investigation of infectious disease (*n* = 12, 9%), suspected drug toxicity (*n* = 3, 2%), and unexplained liver function test abnormalities (*n* = 13, 9%). The median time from ICU admission to liver biopsy was 3 [1–5] days. On the day of liver biopsy, the hemoglobin level was 9 [8–11] g/dL, the platelet count was 89 [50–145] G/L, the International Normalized Ratio (INR) was 2.1 [1.8–2.8], and the fibrinogen level was 2.2 [1.6–3.3] g/L. Overall, 53 (38%) patients received blood products before the biopsy procedure (e-Table 2). Most patients had one or two samples collected during the procedure, while 30 (22%) had three samples or more. The route was transjugular in 97 (70%) patients and percutaneous in 30 (22%) patients; one biopsy was performed during abdominal surgery (missing data, *n* = 11).

The portocaval gradient was measured in 40 (41%) patients biopsied via the transjugular route; the median value was 15 [9–22] mmHg and in three patients a transjugular intrahepatic porto-systemic shunt was implanted during the same procedure.

WHO grade 3–4 bleeding complications developed in 10 (7%) patients, including seven with hemoperitoneum, one with both hemoperitoneum and a subcapsular hepatic hematoma, one with an isolated subcapsular hepatic hematoma, and one with hemothorax (e-Table 3, e-Table 4). Two patients (1.4%) died due to bleeding complications (e-Table 5).

By univariate analysis, factors associated with bleeding were high SOFA and MELD scores, high urea level, and absence of cirrhosis (Table 2).

**Table 2:** Univariate analysis to identify risk factors for bleeding complications

Variables	No bleeding complication (*n* = 129)	Bleeding complication (*n* = 10)	*P* value
Male, n (%)	91 (71)	5 (50)	0.28
Age, years, median [IQR]	52 [44–59]	49 [39–55]	0.50
Body mass index, kg/m², median [IQR]	25 [22–28]	25 [19–27]	0.31
Cirrhosis, n (%)	72 (56)	2 (20)	0.04
SOFA score, median [IQR]	7 [5–10]	10 [6–14]	0.02
SAPS II, median [IQR]	49 [33–60]	56 [50–53]	0.13
MELD score, median [IQR]	26 [22–29]	40 [35–41]	0.02
CLIF-C OF score, median [IQR]	11 [10–13]	11 [10–12]	0.97
**Laboratory parameters on biopsy day**			
Hemoglobin, g/dL, median [IQR]	9 [8–10]	9 [8–10]	0.74
Platelets, G/L, median [IQR]	79 [50–125]	45 [11–112]	0.18
Prothrombin time, %, median [IQR]	37 [29–52]	52 [40–60]	0.16
Serum creatinine, µmol/L, median [IQR]	82 [52–165]	187 [86–295]	0.19
Urea, mmol/L, median [IQR]	8 [5–14]	17 [10–21]	0.02
**Type of liver biopsy**,** n (%)**^**†**^			0.35
Transjugular	91 (71)	6 (60)	
Percutaneous	26 (20)	4 (40)	
Anticoagulant or antiplatelet therapy on the biopsy day, n (%)	19 (15)	0	0.36

SOFA: Sequential Organ Failure Assessment; SAPS II: Simplified Acute Physiology Score version II; MELD: Model for End-stage Liver Disease; CLIF-C OF: Chronic Liver Failure Consortium Organ Failure

^†^ missing data *n* = 11. One patient who had a liver biopsy during laparoscopic surgery was not included in this analysis

### Pathological diagnoses and outcomes

Of the 139 patients, 137 (99%) had evaluable biopsies, which produced 187 diagnoses. The liver parenchyma was normal in 3 (2%) patients. In 83 (60%) patients, the pathological findings were consistent with the diagnostic hypothesis.

**Fig. 2:**
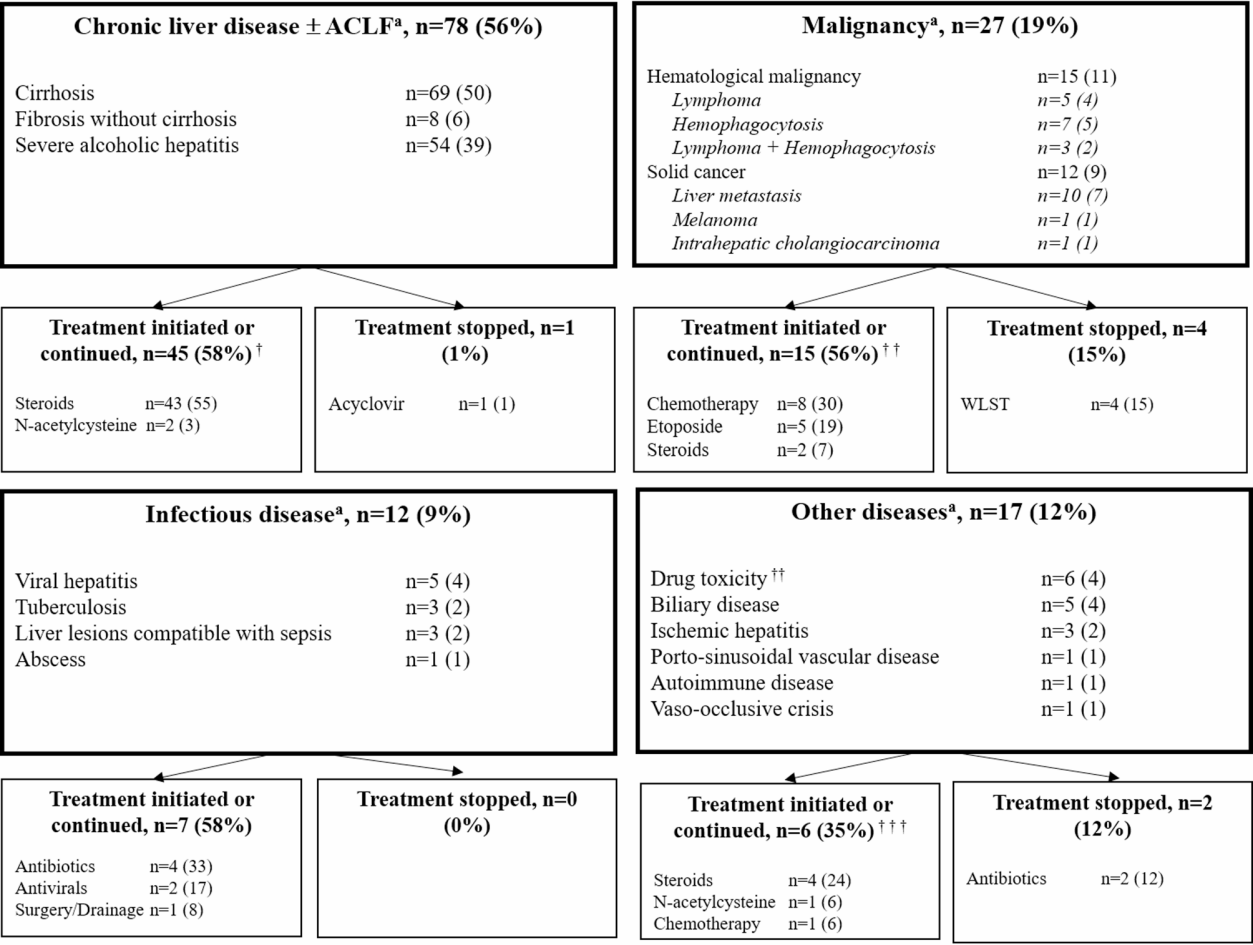
Diagnoses made by liver biopsy and therapeutic decisions ^a^ Some patients had more than one diagnosis ACLF: acute-on-chronic liver failure; WLST: withdrawing life-sustaining therapies ^†^ missing data (*n* = 1), death before pathological result (*n* = 3) ^† †^ missing data (*n* = 1), death before pathological result (*n* = 3) ^† † †^ death before pathological result (*n* = 1)

Figure 2 shows the distribution of the main pathological diagnoses. Chronic and acute-on-chronic liver diseases predominated (*n* = 78, 56%), followed by malignancies (*n* = 27, 19%) and infectious diseases (*n* = 12, 9%). In the remaining 17 (12%) patients, drug toxicity and biliary disease predominated. Severe acute alcoholic hepatitis was the most common acute diagnosis, with the pathological findings confirming a clinical suspicion of this condition in 54/78 (69%) of patients. Among the 26 patients who had a liver biopsy for a suspected malignancy, 9 underwent an ultrasound guided procedure to target a focal lesion of the liver (none of them experienced a bleeding complication).

**Fig. 3:**
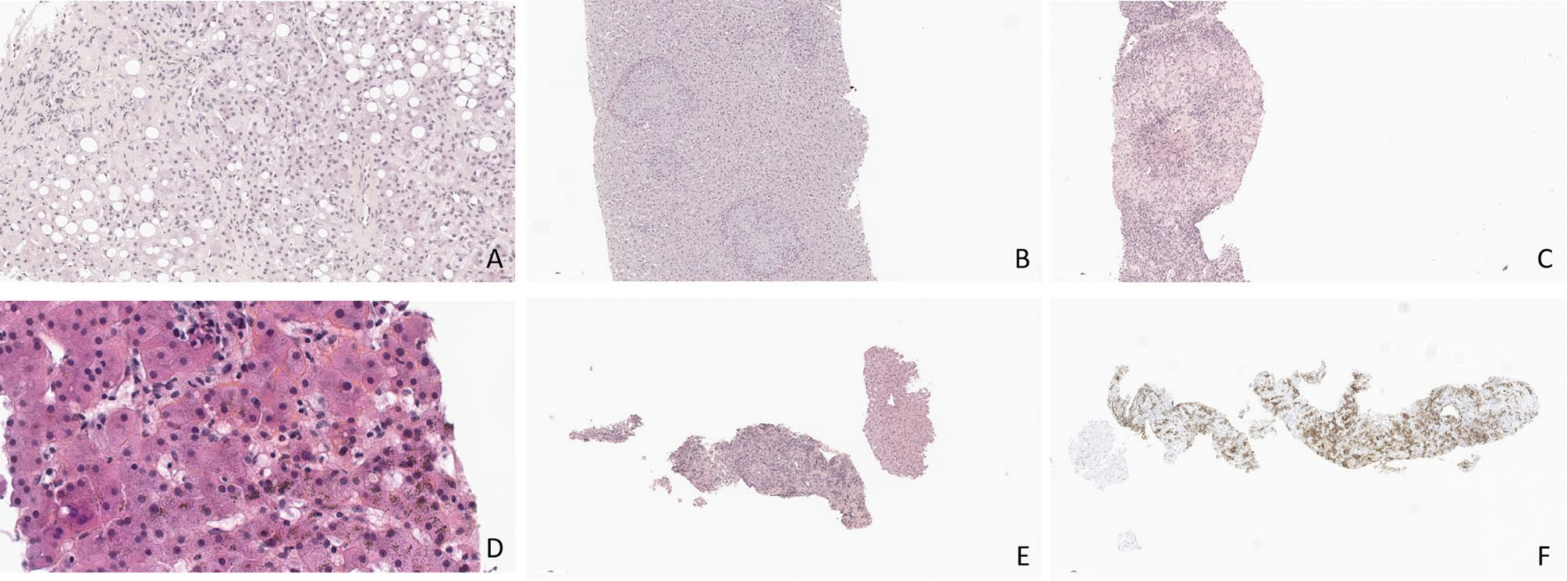
Common histological findings. **(A)** Alcoholic steatohepatitis and cirrhosis with ballooning degeneration of some hepatocytes containing Mallory-Denk bodies and surrounded by mild inflammatory infiltrates made of neutrophil polymorphs (hematein eosin saffron x 40). **(B)** Tuberculosis: necrotic granulomas in the liver parenchyma with peripheral giant cell-and epithelioid-cell reaction (hematein eosin saffron x 20). **(C)** Autoimmune hepatitis, acute clinical presentation: lymphoplasmacytic portal inflammatory infiltrates with interface hepatitis, lobular inflammatory infiltrates, and central bridging necrosis (hematein eosin saffron x 10). **(D)** Reactive hemophagocytosis: Kupffer cells phagocyting erythrocytes and numerous hepatocytes exhibiting hemosiderosis (hematein eosin saffron x 40). **(E)** High-grade B-cell lymphoma with portal infiltration on a transjugular liver biopsy (hematein eosin saffron x 10). **(F)** Immunostaining for CD20 showing malignant lymphoid B cells (in brown) against the fibrous background of the portal tract (x 10)

Overall, the liver biopsy result led to therapeutic decisions in 80 (58%) patients, resulting in the initiation of a new treatment (66/80, 82%), continuation of an empirically initiated treatment (7/80, 9%), or discontinuation of a treatment (7/80, 9%). Day-90 survival was 59% in patients with vs. 51% in those without a therapeutic decision (*P* = 0.25, e-Figure 1). Two patients underwent liver transplantation, 1 year and 1.5 years after the ICU stay, respectively. Figure 3 shows examples of common histological findings.

## Discussion

In this multicenter retrospective observational cohort study, liver biopsies performed in 139 critically ill patients recovered samples containing liver parenchyma in 99% of cases. Liver biopsy proved to be an effective diagnostic tool, demonstrating that the criteria used for patient selection to biopsy were valid. The main diagnoses were ACLF, malignancies, and infections. Importantly, the biopsy results had therapeutic consequences in over half the patients, and the treatment change often consisted in the introduction or continuation of a specific treatment. Finally, 7% of patients experienced bleeding complications, with the risk factors being a worse SOFA score, higher urea level, and absence of cirrhosis. The frequency of bleeding did not differ between transjugular and percutaneous biopsies. Two patients died of bleeding complications.

Although liver test abnormalities are common in ICU patients, the usefulness of liver biopsies in this specific setting had not been studied previously. The high proportion of evaluable liver biopsies obtained by both the transjugular and percutaneous routes is similar to that reported in non-ICU patients in France [24]. In a retrospective study in 77 ICU patients, renal biopsy provided a specific diagnosis in about half the cases and led to treatment modification in only one fifth; two patients experienced severe bleeding requiring embolization [25]. A metaanalysis found that 67 of 232 ICU patients experienced complications after transbronchial biopsy, which provided a diagnosis in 62.9% of cases and led to a treatment change in 49.0% of cases [26]. In our study, the liver biopsy provided a diagnosis in over 90% of cases and the spectrum was broad, including alcohol-related diseases, infections, malignancies, drug toxicities, auto-immune diseases, and rare conditions. The high diagnostic yield suggests that the criteria used to make biopsy decisions were valid. The diagnoses in our cohort vary substantially from those reported in non-ICU patients, in whom chronic viral hepatitis B and C predominate [24]. The proportion of patients in whom the liver biopsy results influenced treatment decisions was considerably higher than previously reported for renal biopsies [25] and slightly higher than for transbronchial lung biopsies [26] in ICU patients.

Bleeding is a major concern when assessing the risk/benefit ratio of invasive procedures in ICU patients in whom thrombocytopenia and hemostasis impairments are common and associated with worse outcomes [27]. In our study, bleeding complications occurred in 10 (7.2%) patients, of whom two died due to the bleeding. This proportion is ten times higher than the rate of serious complications of liver biopsies done outside the ICU [24, 28]. In studies conducted outside the ICU, factors associated with bleeding were older age, female sex, malignancy, low platelet count, high INR, and greater number of passes [29, 30, 31]. In our cohort of critically-ill patients, risk factors for bleeding were severity scores, high urea levels, and absence of cirrhosis. Cirrhosis was not associated with an increased risk of bleeding, in line with two other studies [32, 33]. Such patients may have been carefully selected while other non-cirrhotic conditions, such as hemophagocytic syndrome or haematological malignancies are at risk of bleeding during invasive procedures [34]. Although thrombocytopenia was not associated with bleeding, platelet transfusions were given if deemed necessary before the biopsy. Moreover, our sample size may have been too small to identify a statistically significant difference. The transjugular route for liver biopsy is recommended in patients with contraindications to the percutaneous technique and is considered at low risk of bleeding [35]. However, the biopsy technique was not significantly associated with the risk of bleeding complications in our study.

Our findings demonstrate that liver biopsy provides meaningful information in ICU patients and has an acceptable safety profile. The findings often had immediate therapeutic consequences. However, two points should be made. First, day-90 survival was not significantly different between patients whose biopsy did vs. did not have a therapeutic decision following the liver biopsy. This result may be related to insufficient statistical power. Second, 10 (7%) patients experienced severe bleeding complications, including two who died. Predicting the bleeding risk is extremely challenging. However, our data support particular caution in patients with a high SOFA score, acute kidney injury, and/or no cirrhosis.

Our study provides the first information on the diagnoses provided by liver biopsy in ICU patients and on the therapeutic decisions following those diagnoses. Second, the multicenter design supports the generalizability of the findings. Third, we provide data on the rate of complications of this invasive procedure, which may help intensivists make optimal clinical decisions and accurately inform patients and relatives.

One limitation of our study is the retrospective design, which carries a risk of selection bias and missing data. Second, we may have underestimated the rate of bleeding complications because not all patients underwent liver imaging after the biopsy. However, as we had data on blood product transfusions, any missed bleeding complications would have been minor, with no transfusion requirements. Third, liver biopsy assessment was not centralized. Nonetheless, liver-pathology experts assessed the biopsies in all four participating centers. Fourth, we had no data on the experience of the operator, type of needle used, or imaging-guidance methods for percutaneous biopsies. Such factors may have influenced the diagnostic yield and complication rate. Fifth, all four participating ICUs were in France, potentially limiting the general applicability of our results. Sixth, we included patients over a long period of time during which ICU policies and practices may have changed. Seventh, due to the limited number of bleeding events, we were unable to conduct a multivariate analysis. Finally, day-90 survival was better in the group with a therapeutic decision following the liver biopsy but not significantly so. This result may be ascribable to the limited sample size.

## Conclusion

In the present study in selected ICU patients, liver biopsy provided a wide range of accurate diagnoses and valuable treatment guidance. The most common indications were acute-on-chronic liver disease, malignancies, infections involving the liver, and abnormal liver tests of unknown origin. However, severe bleeding complications did occur and were fatal in two patients. Further research is needed to optimize the safety of liver biopsy in ICU patients.

## Supplementary Information

Below is the link to the electronic supplementary material.

Supplementary Material 1

## Data Availability

The datasets used and/or analyzed during the current study are available from the corresponding author on reasonable request.

## Abbreviations

ACLF: acute-on-chronic liver failure
CLIF-C OF score: Chronic Liver Failure Consortium Organ Failure score
ICD-10: International Statistical Classification of Diseases, 10th Revision
ICU: intensive care unit
INR: International normalized ratio
MELD: Model for End-stage Liver Disease
SAPS II: Simplified Acute Physiology Score version II
SOFA score: Sequential Organ Failure Assessment score
