# Supplementary material for: Diagnostic yield and adverse events of liver biopsy in intensive-care-unit patients: a multicenter retrospective observational cohort study
Source: Ann Intensive Care. 2025 Aug 26;15:123. doi: 10.1186/s13613-025-01533-0 (PMC12379671; doi:10.1186/s13613-025-01533-0)
Supplement: Supplementary file 1 — Supplementary Material 1 [file 13613_2025_1533_MOESM1_ESM.docx]

**SUPPLEMENTARY MATERIAL**

**Safety and Diagnostic Yield of Liver Biopsy in Intensive-Care-Unit Patients: A Multicenter Retrospective Observational Cohort Study**

**Table of contents**

e-Table 1: STROBE Checklist…………………………………………………….…….. p 1

e-Table 2: Transfusion of blood products ….……………………………………..…….. p 4

e-Table 3: Modified WHO Bleeding scale ……….……….…………………………….. p 4

e-Table 4: Characteristics of the ten patients experienced bleeding complications of the liver biopsy………………………………………………………………………………...……p 5

e-Table 5: Characteristics of the two patients who died from bleeding complications of the liver biopsy…………………………………………………………………………………p 6

e-Figure 1: Day-90 survival according to therapeutic decision following the liver biopsy………………………………………………………………………………..……... p 7

**e-Table 1. STROBE Statement—checklist of items that should be included in reports of observational studies**

|  | Item No. | Recommendation | Page number |
| --- | --- | --- | --- |
| Title and abstract | 1 | (*a*) Indicate the study’s design with a commonly used term in the title or the abstract | 1 |
|  |  | (*b*) Provide in the abstract an informative and balanced summary of what was done and what was found | 2 |
| Introduction | | | |
| Background/rationale | 2 | Explain the scientific background and rationale for the investigation being reported | 3 and 4 |
| Objectives | 3 | State specific objectives, including any prespecified hypotheses | 5 |
| Methods | | | |
| Study design | 4 | Present key elements of study design early in the paper | 4 and 5 |
| Setting | 5 | Describe the setting, locations, and relevant dates, including periods of recruitment, exposure, follow-up, and data collection | 4 |
| Participants | 6 | (*a*) *Cohort study*—Give the eligibility criteria, and the sources and methods of selection of participants. Describe methods of follow-up | 4 and 5 |
|  |  | (*b*) *Cohort study*—For matched studies, give matching criteria and number of exposed and unexposed | NA |
| Variables | 7 | Clearly define all outcomes, exposures, predictors, potential confounders, and effect modifiers. Give diagnostic criteria, if applicable | 4 and 5 |
| Data sources/ measurement | 8* | For each variable of interest, give sources of data and details of methods of assessment (measurement). Describe comparability of assessment methods if there is more than one group | 4 and 5 |
| Bias | 9 | Describe any efforts to address potential sources of bias | NA |
| Study size | 10 | Explain how the study size was arrived at | NA |
| Quantitative variables | 11 | Explain how quantitative variables were handled in the analyses. If applicable, describe which groupings were chosen and why | 6 |
| Statistical methods | 12 | (*a*) Describe all statistical methods, including those used to control for confounding | 6 |
|  |  | (*b*) Describe any methods used to examine subgroups and interactions | NA |
|  |  | (*c*) Explain how missing data were addressed | 6 |
|  |  | (*d*) *Cohort study*—If applicable, explain how loss to follow-up was addressed | NA |
|  |  | (*e*) Describe any sensitivity analyses | NA |
| Participants | 13* | (a) Report numbers of individuals at each stage of study—eg numbers potentially eligible, examined for eligibility, confirmed eligible, included in the study, completing follow-up, and analysed | Figure 1 |
|  |  | (b) Give reasons for non-participation at each stage | Figure 1 |
|  |  | (c) Consider use of a flow diagram | Figure 1 |
| Descriptive data | 14* | (a) Give characteristics of study participants (eg demographic, clinical, social) and information on exposures and potential confounders | Table 1 |
|  |  | (b) Indicate number of participants with missing data for each variable of interest | Table 1 |
|  |  | (c) *Cohort study*—Summarise follow-up time (eg, average and total amount) | 6 and 7 |
| Outcome data | 15* | *Cohort study*—Report numbers of outcome events or summary measures over time | 7 and 8 |
| Main results | 16 | (*a*) Give unadjusted estimates and, if applicable, confounder-adjusted estimates and their precision (eg, 95% confidence interval). Make clear which confounders were adjusted for and why they were included | Table 2 |
|  |  | (*b*) Report category boundaries when continuous variables were categorized | NA |
|  |  | (*c*) If relevant, consider translating estimates of relative risk into absolute risk for a meaningful time period | NA |
| Other analyses | 17 | Report other analyses done—eg analyses of subgroups and interactions, and sensitivity analyses | NA |
| Key results | 18 | Summarise key results with reference to study objectives | 8 and 9 |
| Limitations | 19 | Discuss limitations of the study, taking into account sources of potential bias or imprecision. Discuss both direction and magnitude of any potential bias | 11 |
| Interpretation | 20 | Give a cautious overall interpretation of results considering objectives, limitations, multiplicity of analyses, results from similar studies, and other relevant evidence | 9 to 11 |
| Generalisability | 21 | Discuss the generalisability (external validity) of the study results | 10 |
| Other information |  |  |  |
| Funding | 22 | Give the source of funding and the role of the funders for the present study and, if applicable, for the original study on which the present article is based | 13 |

**e-Table 2. Transfusion of blood products^a^**

| **Blood products** | **Before the biopsy**  n=53/139 (38%) | **Within 48 hours after the biopsy**  n = 10 (7%) |
| --- | --- | --- |
| **Platelets** |  |  |
| Patients, n (%) | 19 (14) | 8 (6) |
| Number of units, median [IQR] | 1 [1–2] | 1 [1–3] |
| **Fresh frozen plasma** |  |  |
| Patients, n (%) | 30 (22) | 10 (7) |
| Number of units, median [IQR] | 2 [2–3] | 2,5 [2–5,25] |
| **Red blood cells** |  |  |
| Patients, n (%) | 30 (22) | 8 (6) |
| Number of units, median [IQR] | 1 [1–2] | 5 [3–6] |

^a^Some patients received more than one blood product.

**e-Table 3. Modified WHO Bleeding Scale grades in the 139 patients**

| **Gastrointestinal or intra-abdominal bleeding** | | **Number of patients, n (%)** |
| --- | --- | --- |
| **Grade 1** |  |  |
| **Grade 2** | - Hematemesis - Blackish vomiting - Bleeding in gastric aspirations - Melena - Hematochezia - Bleeding from other abdominal sites |  |
| **Grade 3** | - Any bleeding requiring the transfusion of a maximum of 2 red blood cell packs compared to the patient's usual transfusion support | 1 (0.7) |
| **Grade 4** | - Any bleeding requiring the transfusion of more than 2 red blood cell packs compared with the usual transfusion requirements - Any bleeding requiring mechanical ventilation - Any bleeding requiring surgical management or embolization | 9 (6.5) |

**e-Table 4: Characteristics of the ten patients experienced bleeding complications of the liver biopsy**

|  | **Reason for liver biopsy** | **Biopsy procedure** | **Gender^1^** | **Age**  **(years)** | **Charlson Comorbidity Index** | **SOFA score** | **SAPS II** | **Cirrhosis** | **Vasopressors** | **Invasive mechanical ventilation** | **Renal Replacement Therapy** | **Hemoglobin**  **(g/dL)** | **Platelets**  **(G/L)** | **Prothrombin time**  **(%)** | **Creatinine**  **(µmol/L)** | **Urea**  **(mmol/L)** | **Prophylactic Platelet Transfusion**  **(units)** | **Prophylactic FFP tranfusion**  **(units)** |
| --- | --- | --- | --- | --- | --- | --- | --- | --- | --- | --- | --- | --- | --- | --- | --- | --- | --- | --- |
| **1** | Abnormal liver function tests | Percutaneous | F | 31 | 1 | 19 | 70 | No | Yes | Yes | Yes | 8 | 58 | 79 | 309 | 21 | 1 | 0 |
| **2** | Abnormal liver function tests | Percutaneous | F | 54 | 3 | 5 | NA | No | No | No | No | 7 | 15 | 50 | 86 | 15 | 1 | 0 |
| **3** | Suspected acute alcoholic hepatitis | Transjugular | M | 55 | 3 | 8 | 44 | No | No | No | No | 9 | 19 | 33 | 81 | NA | 0 | 0 |
| **4** | Suspected hemophagocytosis | Percutaneous | F | 64 | 4 | 4 | 57 | No | No | No | No | 11 | 176 | 95 | 63 | 6 | 0 | 0 |
| **5** | Suspected acute alcoholic hepatitis | Transjugular | M | 48 | 7 | 13 | 50 | Yes | Yes | Yes | Yes | 9 | 8 | 36 | 328 | 28 | 1 | 2 |
| **6** | Suspected acute alcoholic hepatitis | Transjugular | M | 46 | 3 | 14 | 43 | No | Yes | Yes | No | 7 | 32 | 24 | 187 | 20 |  |  |
| **7** | Suspected hemophagocytosis | Transjugular | F | 37 | 1 | 6 | 52 | No | Yes | Yes | Yes | 9 | 143 | 54 | 295 | 10 | 0 | 0 |
| **8** | Suspected acute alcoholic hepatitis | Transjugular | M | 32 | 3 | 12 | 63 | No | No | Yes | No | 10 | 8 | 50 | 274 | 6 | 0 | 0 |
| **9** | Suspected acute alcoholic hepatitis | Transjugular | M | 49 | 4 | 16 | 96 | Yes | No | Yes | Yes | 8 | 115 | 58 | NA | 40 | 0 | 3 |
| **10** | Suspected autoimmune hepatitis | Percutaneous | F | 71 | 4 | 7 | 56 | No | Yes | Yes | Yes | 12 | 104 | 60 | 115 | 17 | 0 | 0 |

F: Female; FFP: Fresh Frozen Plasma; M: Male; SAPS II: Simplified Acute Physiology Score version II; SOFA: Sequential Organ Failure Assessment

**e-Table 5. Characteristics of the two patients who died following the liver biopsy**

|  | **Patient 1** | **Patient 2** |
| --- | --- | --- |
| **Age, years** | 54 | 49 |
| **Underlying disease** | Lymphoma | Cirrhosis (CHILD C11) |
| **SAPS II** | Not recorded | 96 |
| **Reason for the biopsy** | Abnormal liver tests of unknown origin (cholestatic hepatitis) | Clinical suspicion of severe acute alcoholic hepatitis |
| **Type of biopsy** | Percutaneous | Transjugular |
| **Coagulation parameters on the day of liver biopsy** |  |  |
| Prothrombin time (%) | 50 | 58 |
| Platelet count (G/L) | 15 | 115 |
| **Transfusion of blood products before liver biopsy** | One platelet transfusion | Three fresh frozen plasma transfusions |
| **Treatment with platelet inhibitor or anticoagulant** | No | No |

**e-Figure 1. Day-90 survival according to therapeutic decision following the liver biopsy**

**
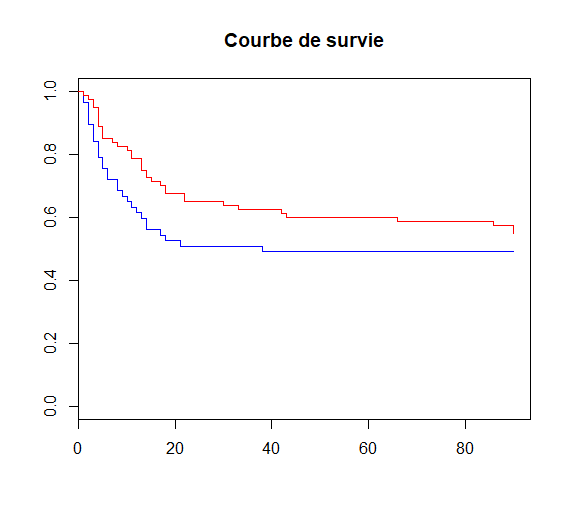
**

**Days from ICU admission**

**Overall Survival (%)**

p = 0.25

Red line: therapeutic decision; Blue line: no therapeutic decision

Day-90 survival was not significantly different between the groups with vs. without a therapeutic decision following the liver biopsy (59% and 51%, respectively; *P*=0.25).
